# The Impact of the Coronavirus Disease - 19 Pandemic on the Clinical Characteristics and Treatment of Adult Patients with Acute Appendicitis

**DOI:** 10.3389/fsurg.2022.878534

**Published:** 2022-03-31

**Authors:** Sanghyun An, Hae-Rim Kim, Sungwoo Jang, Kwangmin Kim

**Affiliations:** ^1^ Department of Surgery, Yonsei University Wonju College of Medicine, Wonju, Korea; ^2^ College of Natural Science, School of Statistics, University of Seoul, Seoul, Korea

**Keywords:** COVID-19, coronavirus, pandemic, appendicitis, appendectomy

## Abstract

**Purpose:**

This study aimed to investigate the characteristics, severity, and treatment of adult patients with acute appendicitis in Korea over a 2-year period during the coronavirus disease (COVID-19) pandemic compared to those before the pandemic. We also investigated whether there were any changes in clinical characteristics of acute appendicitis before and after vaccination against the coronavirus.

**Methods:**

We retrospectively reviewed the medical records of patients who were diagnosed with acute appendicitis at our institution between March 1, 2019, and August 31, 2021. We divided the patients into three groups (pre-pandemic, before vaccination, and after vaccination) and analyzed the clinical outcomes.

**Results:**

The time from symptom onset to hospital arrival and the time from symptom onset to operation increased during the COVID-19 pandemic period compared to the pre-pandemic period. The rate of complicated appendicitis during the pandemic was higher than that before the pandemic. In addition, the number of new daily cases showed a positive correlation with the time from symptom onset to hospital arrival (OR, 0.03; 95% CI, 0.02 to 0.04; *P* < 0.001) and complicated appendicitis (OR, 1.002; 95% CI, 1.001–1.002; *P* = 0.0017). The vaccination rate showed a negative correlation with the time from symptom onset to hospital arrival (OR, −2.26; 95% CI, −3.42 to −1.11; *P* < 0.001) and complicated appendicitis (OR, 0.915; 95% CI, 0.84 to 0.996; *P* = 0.0404).

**Conclusions:**

Employing hospital-wide efforts, such as screening by rapid PCR testing, to avoid further time delays, and nationwide efforts, such as vaccination, to shorten the time from symptom onset to hospital arrival, are necessary to maintain the quality of treatment of acute appendicitis during an infectious disease pandemic.

## Introduction

Coronavirus disease (COVID-19) is an infectious disease caused by severe acute respiratory syndrome coronavirus 2 (SARS-CoV-2) and was first reported in Wuhan, Hubei, China in December 2019 (1). The virus, which can spread through direct contact, aerosols, and airborne droplets, mainly affects the upper respiratory tract and can cause pneumonia in severe cases. This highly contagious virus spread all over the world in a short time, leading the World Health Organization to declare COVID-19 a pandemic on March 11, 2020 (2). With the spread of COVID-19, many countries declared a state of emergency and instituted partial or full restrictions on social activities (3). Furthermore, this global social atmosphere impacted the medical field as non-urgent medical services were recommended to be postponed (4). Some studies have reported a significant reduction in the number of patient visits to hospitals, including emergency clinics, during the pandemic (5, 6). Particularly, in the surgery part, non-urgent elective operations are often delayed or canceled to reduce the overburden of the healthcare system and reduce the risk of COVID-19 infection. Aerosolized droplets generated when making pneumoperitoneum in minimally-invasive surgeries, such as laparoscopy and robotic surgeries, which have taken up a large part in recent decades, can increase the risk of COVID-19 infection and threaten surgeons and other healthcare workers (7, 8). Moreover, several studies have demonstrated that patients with COVID-19 had increased postoperative morbidity, including pulmonary complications, and high mortality (9, 10). For these reasons, various guidelines have recommended delaying non-urgent elective surgery and proceeding with conservative treatment for diseases that can be treated conservatively (11). However, there are still inevitable and emergent clinical situations that require immediate treatment.

Acute appendicitis is one of the most common acute abdominal diseases requiring emergency treatment, and the lifetime risk of developing acute appendicitis is approximately 7–8% worldwide (12, 13). Emergency appendectomy is the treatment of choice, but there is still debate about its suitability in this pandemic era because several articles have reported that non-surgical management, such as antibiotic therapy, is non-inferior to appendectomy (14, 15). In addition, it has been reported that initial non-surgical management, such as antibiotics, with or without percutaneous drainage followed by interval appendectomy in complicated cases such as periappendiceal abscess is effective and reduces postoperative complications compared to immediate appendectomy (16). Moreover, a change in the main treatment strategy has been observed in some countries. In the UK, the antibiotic-first pathway has been accepted and implemented as the main treatment strategy for acute appendicitis in the era of the COVID-19 pandemic (17). Other changes associated with the management of appendicitis during the COVID-19 pandemic were also reported in several studies; the number of patients visiting the hospital for acute appendicitis decreased and the time from symptom onset to hospital arrival (TSH) increased, resulting in an increase in complicated cases (18–20). Further, the risk of complications such as perforation increased as the time from symptom onset to treatment became longer than 24 h (21). However, most of these studies are reports on the initial situation of the pandemic outbreak and do not reflect the post-vaccination changes or the current ongoing situation due to the mutant virus.

Therefore, this study aimed to investigate the changes in the characteristics, severity, and treatment of patients with acute appendicitis in Korea for 2 years following the advent of the COVID-19 pandemic compared to those during the pre-pandemic period. We also investigated the impact of the vaccinations against the coronavirus on the characteristics, severity, and treatment of patients with acute appendicitis. Furthermore, we investigated the factors associated with the outcomes.

## Methods

### Study Population

**Figure 1:**
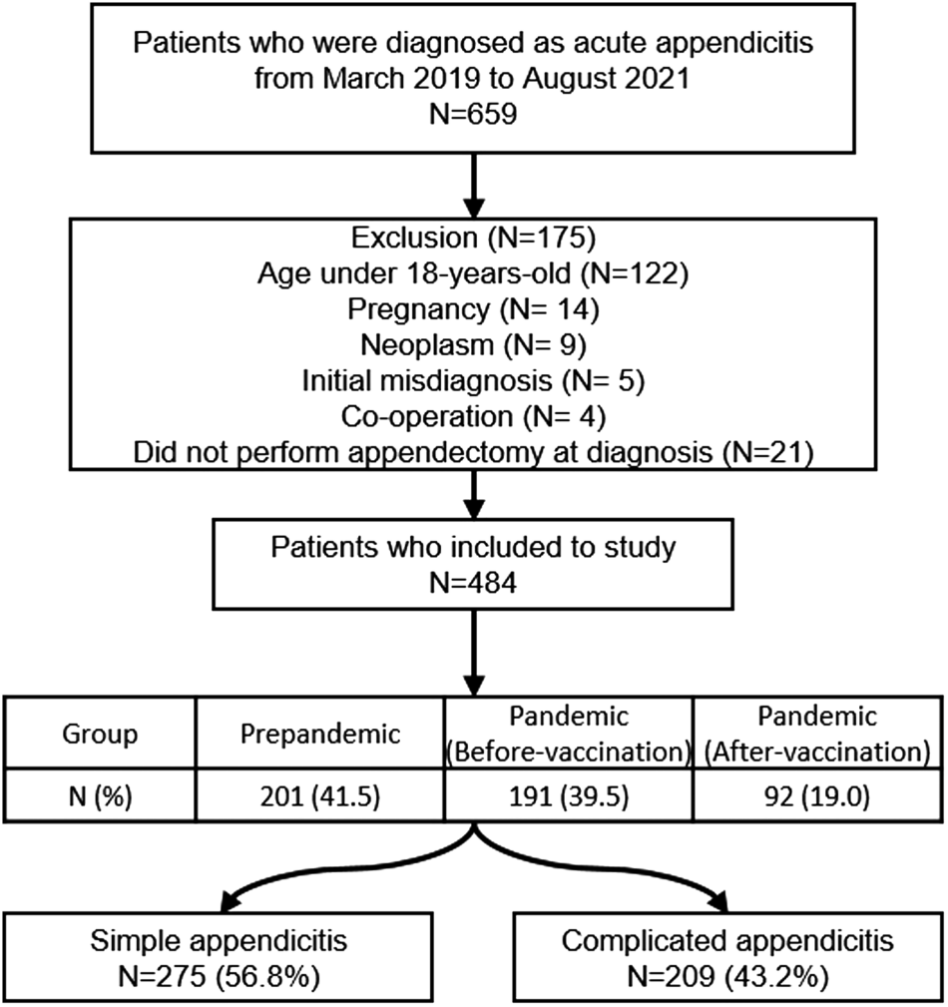
Patients included in the study.

We retrospectively reviewed the medical records of patients who were diagnosed with acute appendicitis at Yonsei University Wonju Severance Christian Hospital, Republic of Korea, from March 1, 2019, to August 31, 2021. During this period, 659 patients were diagnosed with acute appendicitis in the emergency clinic. Of these, we excluded patients who were younger than 18 years, pregnant, diagnosed with benign or malignant neoplasm on pathological examination, referred from other departments or hospitals (e.g., misdiagnosis) and underwent combined operation and those who did not undergo appendectomy at initial diagnosis. Finally, 484 participants were included in the study (Figure 1). This study was approved by the Institutional Review Board of Yonsei University Wonju Severance Christian Hospital (IRB no. CR321177). Because this study was conducted retrospectively, informed consent was waived.

### COVID-19 Pandemic and Vaccination in the Republic of Korea

**Figure 2:**
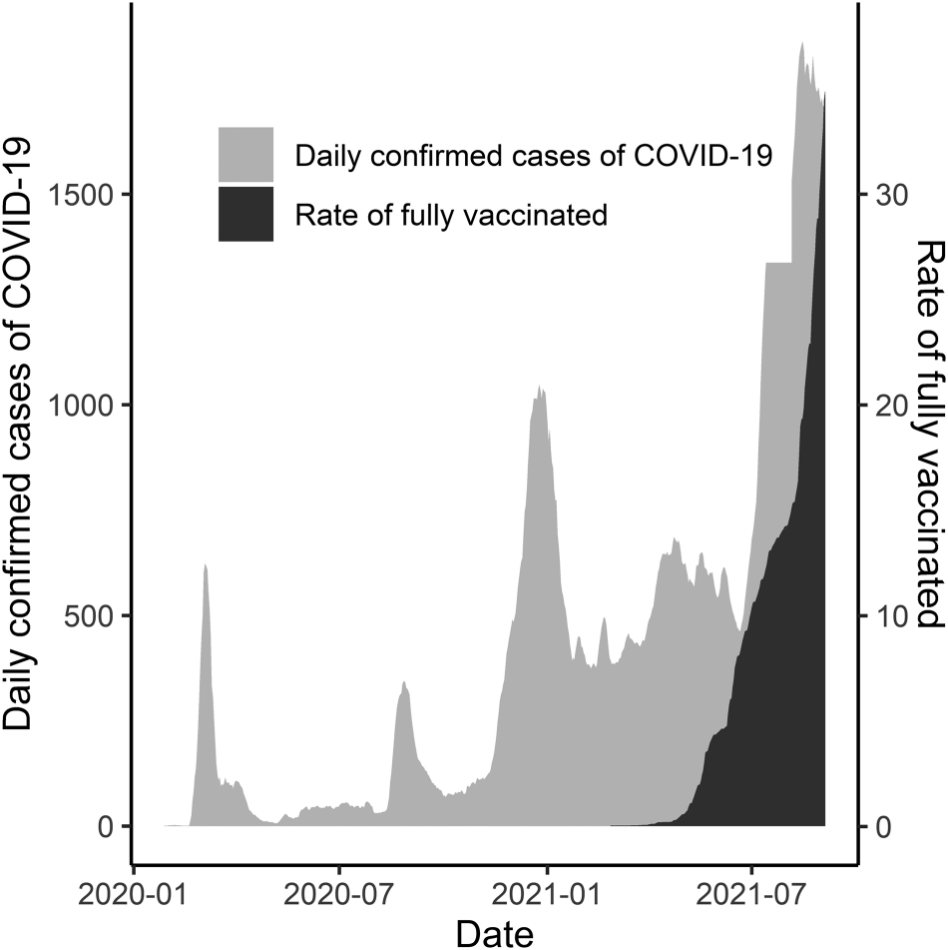
Daily confirmed cases of COVID-19 and percentage of fully vaccinated individuals until August 31, 2021.

The first case of COVID-19 in the Republic of Korea occurred on January 20, 2020, and the first outbreak started on February 18, 2020. Since then, the number of confirmed cases of COVID-19 has continued to increase; as of August 31, 2021, the cumulative number of confirmed cases was 253,445. In Korea, vaccination for COVID-19 began on February 26, 2021, and the percentage of fully vaccinated individuals on August 31, 2021, was 31.1% (Figure 2) (22). In this study, the period before the first outbreak (February 18, 2020) has been defined as pre-pandemic (PP) and that after the first outbreak as pandemic (P). We further divided the pandemic group into the before-vaccination period (P-BV) and after-vaccination period (P-AV) based on the starting date of vaccinations in Korea. We defined the completion of vaccination as up to the second vaccination.

### Data Collection

Demographic characteristics of the patients, including age, sex, pulse rate, body temperature at arrival, length of stay after operation (LOS), operation time, duration of antibiotic use, TSH, time from hospital arrival to operation (THO), time from symptom onset to operation (TSO), American Society of Anesthesiologists physical status (ASA) score, medical history, type of operation, whether laparotomy or laparoscopy was performed, whether open conversion during laparoscopy was performed, whether the drain was inserted after the operation, radiological findings (acute inflammation, periappendiceal abscess with localized peritonitis, generalized peritonitis), pathological findings (acute inflammation, suppurative, gangrenous, perforation, abscess), postoperative complications, and re-visit to the emergency department or re-admission within 30 days after operation, were obtained from medical records. Initial laboratory results including white blood cell counts (WBC), hemoglobin (Hb), platelet count (PLT), neutrophil count, lymphocyte count, delta neutrophil index (DNI), and C-reactive protein (CRP) levels were also obtained. The type of operation performed was categorized as follows: appendectomy, partial cecectomy, ileocecectomy, and right hemicolectomy. To analyze the correlation between the transmission rate of COVID-19 and the clinical features of appendicitis, the number of daily confirmed cases of COVID-19 and the vaccination rates on the day of hospitalization were also investigated using data from the Korea Centers for Disease Control and Prevention.

### Definitions

Appendicitis is divided into simple appendicitis and complicated appendicitis. In our study, complicated appendicitis was defined as one or more of the following three findings: 1. Cases that underwent additional bowel resection (e.g., partial cecectomy, ileocecectomy, or right hemicolectomy); 2. Presence of pathological findings such as gangrenous inflammation, perforation, or periappendiceal abscess; and 3. Presence of radiological findings such as localized peritonitis with abscess formation or generalized peritonitis.

### Statistical Analysis

The patients were classified into three groups based on their date of visit to the emergency clinic, namely, pre-pandemic (PP), pandemic (P-BV), or pandemic (P-AV). Continuous variables are expressed as median (interquartile range [IQR]), and categorical variables are expressed as frequencies and percentages. Continuous data were tested for normal distribution using the Shapiro-Wilk test and compared using the analysis of variance (ANOVA) test or the Kruskal-Wallis test, as appropriate. When a significant difference between the three groups was found in the ANOVA test or the Kruskal-Wallis test, a post-hoc test was performed using the Benjamini-Yekutieli correction. Categorical variables were also tested for normality and compared using the chi-square test and Fisher’s exact test, as appropriate. Binary logistic regression analysis was performed to identify factors associated with complicated appendicitis. Univariate and multivariate analyses were performed, and variables were selected automatically using backward stepwise selection. Linear regression analysis was also performed with TSH, THO, and TSO as dependent variables. Similarly, independent variables were selected for backward stepwise selection. Statistical significance was set at *p *< 0.05, except Dunn’s post-hoc test by Benjamini-Yekuteili adjustment. In the post-hoc test, the null hypothesis was rejected when *p* < 0.05/2. Statistical analysis was performed using R statistical software (version 4.1.0; R Foundation for Statistical Computing, Vienna, Austria).

## Results

### Comparison of Patient Characteristics Between the Three Groups

**Figure 3:**
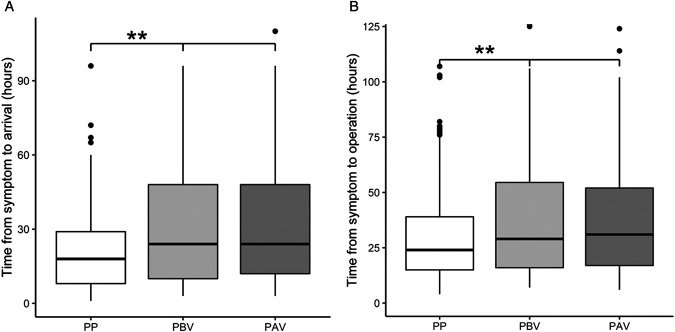
(**A**) Time from symptom to arrival between the three groups. (**B**) Time from symptom to operation between the three groups. ** statistically significant between two groups in post-hoc analysis. PP, pre-pandemic; PBV, pandemic (before vaccination); PAV, pandemic (after vaccination).

Among the 484 patients diagnosed with acute appendicitis who underwent emergency surgery, 201 and 283 patients were in the pre-pandemic and pandemic groups, respectively. In the pandemic group, there were 191 patients in the pre-vaccination period and 92 patients in the post-vaccination period. The median age was 51 years (IQR 38–63), and 102 patients were male (50.7%). General characteristics such as age, sex, ASA score, medical history, and abdominal operation history did not show significant statistical differences among the three groups. No statistical differences were observed in body temperature or pulse rate between the three groups at the time of arrival. Laboratory test results at the time of arrival showed that the DNI value was significantly higher in the pandemic group than in the pre-pandemic group; the other laboratory parameters did not differ among the three groups. In the post-hoc analysis, the DNI value of the P-AV group was significantly higher than that of the PP and P-BV groups (*P* = 0.037 and *P* = 0.032, respectively); there were no differences between the PP and P-BV groups. The median TSH during the pandemic was 24 h, significantly longer than the 18 h of the pre-pandemic group (*P* < 0.001). However, the THO did not differ between the three groups. The TSO was 24 h in the pre-pandemic group, 29 h in the P-BV group, and 31 h in the P-AV group, showing a significant difference among the three groups (Table 1). In the post-hoc analysis, the median TSH and median TSO did not differ between the P-BV and P-AV groups (Figures 3A,B). Hospital LOS after surgery did not differ between the three groups (Table 2).

**Table 1:** Demographic characteristics of enrolled patients.

	Pre-pandemic	Pandemic (BV)	Pandemic (AV)	*P* value
	(*N* = 201)	(*N* = 191)	(*N* = 92)	
Age	51.0 [38.0; 63.0]	49.0 [32.0; 63.0]	52.0 [36.5; 63.5]	0.626
Sex				0.770
Male	102 (50.7%)	103 (53.9%)	50 (54.3%)	
Female	99 (49.3%)	88 (46.1%)	42 (45.7%)	
ASA physical status				0.501
1	58 (28.9)	51 (26.7)	23 (25.0)	
2	104 (51.7)	114 (59.7)	50 (54.3)	
3	38 (18.9)	26 (13.6)	19 (20.7)	
4	1 (0.5)	0 (0.0)	0 (0.0)	
Medical history	71 (35.3)	82 (42.9)	36 (39.1)	0.304
Abdominal operation history	23 (11.4)	22 (11.5)	7 (7.6)	0.559
Body temperature (°C)	37.0 [36.7; 37.5]	37.1 [36.6; 37.7]	37.2 [36.8; 37.6]	0.686
Pulse rate (beats/minute)	83.0 [71.0; 95.0]	86.0 [76.0; 98.5]	86.0 [77.0; 98.0]	0.148
White blood cell count (10^9^/L)	11.0 [8.4; 14.1]	12.4 [9.0; 15.1]	12.3 [9.8; 15.1]	0.142
Hemoglobin (g/dL)	14.0 [13.0; 15.1]	14.2 [13.0; 15.3]	14.0 [13.1; 14.8]	0.564
Platelet count (10^11^/L)	2.3 [2.0; 2.7]	2.4 [2.0; 2.9]	2.3 [1.9; 2.7]	0.564
Neutrophil count (10^9^/L)	8.7 [6.2; 12.2]	9.9 [6.6; 13.0]	9.9 [7.5; 12.5]	0.113
Lymphocyte count (10^9^/L)	1.3 [0.9; 1.9]	1.4 [0.9; 1.9]	1.3 [0.9; 1.8]	0.851
Delta neutrophil index (%)	0.3 [0.0; 2.2]	0.4 [0.0; 2.2]	1.2 [0.2; 2.5]	0.034
C-reactive protein (mg/dL)	2.9 [0.6; 10.2]	3.5 [0.4; 9.8]	2.6 [0.6; 8.4]	0.768
Duration of antibiotics use	4.0 [3.0; 6.0]	4.0 [2.0; 7.0]	4.0 [2.0; 5.5]	0.069
Time from symptom onset to hospital visit (hours)	18.0 [8.0; 29.0]	24.0 [10.0; 48.0]	24.0 [12.0; 48.0]	<0.001
Time from hospital visit to operation (hours)	5.0 [4.0; 8.0]	5.0 [4.0; 7.0]	5.0 [4.0; 6.0]	0.068
Time from symptom onset to operation (hours)	24.0 [15.0; 39.0]	29.0 [16.0; 54.5]	31.0 [17.0; 52.0]	0.005
LOS after operation (days)	3.0 [2.0; 6.0]	4.0 [2.0; 7.0]	3.0 [2.0; 5.0]	0.412

*Data expressed as median [first quartile; third quartile] for continuous variables and* *n* (%)/ *for nominal variables, BV, Before-vaccination period; AV, After-vaccination period; Hospital LOS, Hospital length of stay*.

**Table 2:** *P* values of Kruskal-Wallis rank sum test and Dunn’s Post-hoc test by Benjamini-Yekuteili adjustment.

Variable	Kruskal-Wallis rank sum test	Dunn’s Post-hoc test (Benjamini-Yekuteili adjustment)		
		PP vs PBV	PP vs PAV	PBV vs PAV
Delta neutrophil index	0.034	0.757	0.037	0.032
Time from symptom to arrival	<0.001	0.001	0.007	0.886
Time from arrival to operation	0.068	0.563	0.062	0.086
Time from symptom to operation	0.005	0.007	0.028	0.832

*In Dunn’s post-hoc test by Benjamini-Yekuteili adjustment, the null hypothesis can be rejected when* *p* < *0.05/2, PP, Pre-pandemic; PBV, Before-vaccination period in pandemic; PAV, After-vaccination period in pandemic*.

### Comparison of Perioperative Characteristics Between the Three Groups

**Figure 4:**
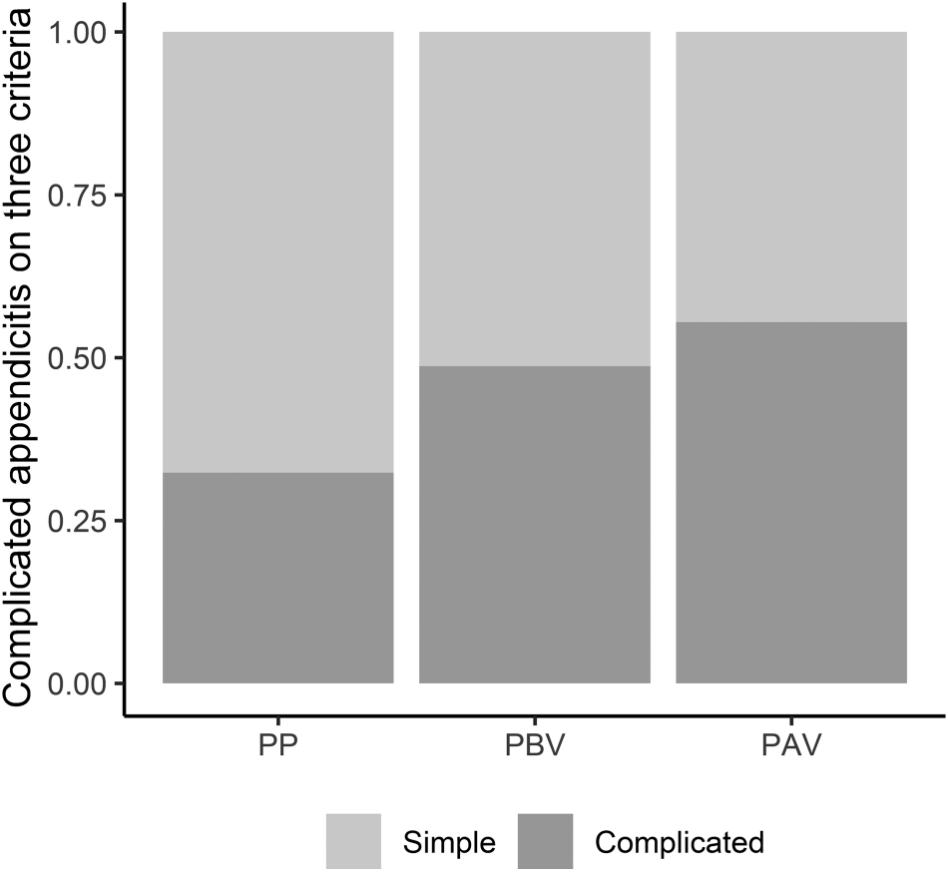
The proportion of complicated appendicitis on three criteria. PP, pre-pandemic; PBV, pandemic (before vaccination); PAV, pandemic (after vaccination).

The type and method of operation did not show a significant difference among the three groups; the operation time was the longest in the pre-pandemic group, with a median value of 50 min. The proportion of complicated appendicitis in pathologic report, including gangrenous change, perforation, or periappendiceal abscess, was significantly higher in the pandemic group than in the pre-pandemic group (*P* = 0.003). In terms of radiological findings, the proportion of complicated cases, such as those with abscess formation or peritonitis, was significantly higher in the pandemic group than in the pre-pandemic group (*P* = 0.004). The proportion of complicated appendicitis according to our definition using operative, pathologic, and radiologic findings was significantly higher in the pandemic group than in the pre-pandemic group (*P* = 0.003) (Figure 4). There was no difference between the three groups in the number of cases with sepsis that required treatment in the intensive care unit, and there was no difference in the incidence of postoperative complications between the three groups. The re-hospitalization rate within 30 days after discharge differed between the three groups, with the P-AV group showing the highest value than the other groups (Table 3).

**Table 3:** Therapeutic characteristics of enrolled patients.

	Pre-pandemic	Pandemic (BV)	Pandemic (AV)	*P* value
	(*N* = 201)	(*N* = 191)	(*N* = 92)	
Operation type (range)				0.727
Appendectomy	170 (84.6)	157 (82.2)	76 (82.6)	
Partial cecectomy	29 (14.4)	28 (14.7)	15 (16.3)	
Ileocecectomy	1 (0.5)	3 (1.6)	1 (1.1)	
Right hemicolectomy	1 (0.5)	3 (1.6)	0 (0.0)	
Operation type (method)				0.149
Laparoscopy	191 (95.0)	170 (89.0)	87 (94.6)	
Open conversion	5 (2.5)	13 (6.8)	2 (2.2)	
Laparotomy	5 (2.5)	8 (4.2)	3 (3.3)	
Operation time (minutes)	50.0 [35.0; 65.0]	40.0 [30.0; 55.0]	45.0 [25.0; 65.0]	0.015
Pathological type				0.031
Normal appendix	4 (2.0)	2 (1.0)	3 (3.3)	
Simple appendicitis	48 (23.9)	24 (12.6)	15 (16.3)	
Suppurative appendicitis	95 (47.3)	84 (44.0)	37 (40.2)	
Gangrenous appendicitis	4 (2.0)	4 (2.1)	4 (4.3)	
Perforated appendicitis	38 (18.9)	51 (26.7)	22 (23.9)	
Periappendiceal abscess	12 (6.0)	26 (13.6)	11 (12.0)	
Radiological findings				<0.001
Normal appendix	7 (3.5)	3 (1.6)	2 (2.2)	
Simple appendicitis	153 (76.1)	129 (67.5)	55 (59.8)	
Abscess, localized peritonitis	37 (18.4)	51 (26.7)	14 (15.2)	
Generalized peritonitis	4 (2.0)	8 (4.2)	21 (22.8)	
Complicated appendicitis on three criteria	65 (32.3)	93 (48.7)	51 (55.4)	0.003
Criteria for complicated appendicitis				
Operation type	31 (15.4)	34 (17.8)	16 (17.4)	0.806
Pathological type	54 (26.9)	81 (42.4)	37 (40.2)	0.003
Radiological type	41 (20.4)	59 (30.9)	35 (38.0)	0.004
Numbers that meet the criteria				<0.001
1	22 (10.9)	37 (19.4)	23 (25.0)	
2	25 (12.4)	31 (16.2)	19 (20.7)	
3	18 (9.0)	25 (13.1)	9 (9.8)	
Admission to ICU	9 (4.5)	12 (6.3)	4 (4.3)	0.668
Complication				0.490
Surgical site infection	19 (9.5)	26 (13.6)	13 (14.1)	
Intra-abdominal abscess	4 (2.0)	5 (2.6)	2 (2.2)	
Postoperative ileus	11 (5.5)	17 (8.9)	3 (3.3)	
Pulmonary complication	2 (1.0)	4 (2.1)	0 (0.0)	
Enteritis	1 (0.5)	3 (1.6)	0 (0.0)	
Myocardial infarction	0 (0.0)	0 (0.0)	1 (1.1)	
Re-admission after discharge	5 (2.5)	4 (2.1)	7 (7.7)	0.035

*Data expressed as n (%) for nominal variables, BV; Before-vaccination period, AV; After-vaccination period, ICU; Intensive care unit*.

### Factors Associated with the Time from Symptom Onset to Hospital Arrival

In linear logistic regression analysis, age, WBC, PLT, and CRP were found to be factors associated with the time from symptom onset to hospital arrival, while multivariate analysis showed that age, daily new cases, vaccination rate, WBC count, PLT, and CRP were independently related factors for the time from symptom onset to hospital arrival. The number of daily new cases showed a positive correlation with the time from symptom onset to hospital arrival (OR, 0.03; 95% CI, 0.02 to 0.04; *P* < 0.001), and the vaccination rate showed a negative correlation with the time from symptom onset to hospital arrival (OR, −2.26; 95% CI, −3.42 to −1.11; *P* < 0.001).

### Factors Associated with the Time from Symptom Onset to Operation

Age, daily new cases, vaccination rate, WBC count, PLT count, and CRP level were independently associated with the time from symptom onset to operation in multivariate linear regression analysis. The number of daily new cases showed a positive correlation with the time from symptom onset to operation (OR, 0.03; 95% CI, 0.01 to 0.04; *P* < 0.001), and the vaccination rate showed a negative correlation with the time from symptom onset to operation (OR, −2.26; 95% CI, −3.41 to −1.1; *P* < 0.001) (Table 4).

**Table 4:** Linear regression for time from symptom onset to hospitalization and time from symptom onset to operation.

	Time from symptom onset to hospitalization				Time from symptom onset to operation			
	Univariate		Multivariate		Univariate		Multivariate	
	OR (95% CI)	*P* value	OR (95% CI)	*P* value	OR (95% CI)	*P* value	OR (95% CI)	*P* value
Age	0.39 (0.21, 0.57)	<0.001	0.25 (0.07, 0.42)	0.006	0.39 (0.21, 0.56)	<0.001	0.25 (0.08, 0.43)	0.005
Sex								
Male	(Reference)		(Reference)		(Reference)		(Reference)	
Female	−1.19 (−8.07, 5.68)	0.734	−4.26 (−10.59, 2.07)	0.188	−1.21 (−8.08, 5.65)	0.729	−4.29 (−10.64, 2.06)	0.186
Daily new cases	0.00 (0.00, 0.01)	0.254	0.03 (0.02, 0.04)	<0.001	0.00 (0.00, 0.01)	0.316	0.03 (0.01, 0.04)	<0.001
Vaccinated rate	−0.20 (−0.89, 0.50)	0.578	−2.26 (−3.42, −1.11)	<0.001	−0.24 (−0.93, 0.45)	0.496	−2.26 (−3.41, −1.1)	<0.001
WBC count	−0.94 (−1.78, −0.10)	0.028	−1.53 (−2.32, −0.74)	<0.001	−0.89 (−1.73, −0.05)	0.038	−1.47 (−2.26, −0.67)	<0.001
Platelet count	0.07 (0.02, 0.12)	0.004	0.13 (0.08, 0.17)	<0.001	0.07 (0.02, 0.12)	0.004	0.13 (0.08, 0.17)	<0.001
CRP	1.49 (1.09, 1.88)	<0.001	1.51 (1.11, 1.90)	<0.001	1.44 (1.05, 1.84)	<0.001	1.45 (1.05, 1.85)	<0.001

*OR, Odd ratio; CI, Confidence interval; WBC, White blood cells; CRP, C-reactive protein.*

### Factors Associated with Complicated Appendicitis

In the binary logistic regression analysis performed to analyze factors associated with complicated appendicitis, age, the number of daily new cases, DNI, and CRP were identified as relevant factors in the univariate analysis. In the multivariate analysis, age, number of daily new cases, vaccination rate, DNI, and CRP were identified as independent relevant factors for complicated appendicitis. The number of daily new cases showed a positive correlation with complicated appendicitis (OR, 1.002; 95% CI, 1.001–1.002; *P* = 0.0017). The vaccination rate was negatively correlated with complicated appendicitis (odds ratio [OR], 0.915; 95% CI, 0.84 to 0.996; *P* = 0.0404) (Table 5).

**Table 5:** Univariate & Multivariate binary logistic regression for complicated appendicitis and Ordinal regression for numbers that meet the criteria for complicated appendicitis.

	Univariate		Multivariate	
	Binary logistic regression			
	OR (95% CI)	*P* value	OR (95% CI)	*P* value
Age	1.035 (1.024,1.046)	<0.001	1.024 (1.011,1.037)	<0.001
Sex				
Male	(Reference)		(Reference)	
Female	0.868 (0.604,1.246)	0.443	0.857 (0.545,1.347)	0.504
Daily new cases	1.001 (1.000,1.001)	0.022	1.002 (1.001,1.002)	0.0017
Vaccinated rate	1.024 (0.987,1.062)	0.2018	0.915 (0.84,0.996)	0.0404
WBC count	1.017 (0.973,1.063)	0.4594	1.01 (0.952,1.072)	0.7318
Platelet count	0.999 (0.996,1.001)	0.3162	1.003 (0.999,1.006)	0.1304
DNI	1.293 (1.182,1.416)	<0.001	1.115 (1.012,1.228)	0.0272
CRP	1.188 (1.144,1.234)	<0.001	1.159 (1.114,1.206)	<0.001
Hosmer & Lemeshow goodness of fit test			*P* = 0.509	
AUC of ROC curve (95% CI)		0.828 (0.791–0.866)		

*OR, Odd ratio; CI, Confidence interval; WBC, White blood cells; DNI, Delta neutrophil index; CRP, C-reactive protein; AUC, Area under curve; ROC, Receiver operating characteristic*.

## Discussion

In the COVID-19 era, several variables of patients with acute appendicitis worsened compared to those in the pre-pandemic era. Among them, TSH and TSO first increased during the COVID-19 pandemic period compared to the pre-pandemic period in this study. In addition, we found no significant differences in postoperative complications despite a delay in TSH and TSO. Therefore, it is not clear whether the delay itself affects postoperative complications.

Similar to our findings, other studies have also reported a prolonged TSH in the pandemic era (18, 23). Zheng et al. (18) suggested that the reason for the delay in TSH was that patients were concerned about COVID-19 transmission at the hospital. Another study also explained that the reluctance to visit the ER was due to the government’s strong social distancing policy and patients’ fear of an in-hospital coronavirus infection (24). These reasons may lead patients to not see a doctor when they have abdominal pain and other early symptoms of acute appendicitis. The TSO may be prolonged for the same reasons as the TSH because the TSO in the pandemic era was not prolonged compared to that in the pre-pandemic era, as observed in this study.

Our study also showed a rise in cases of complicated appendicitis during the pandemic compared to the pre-pandemic period. This may be associated with delayed TSH. In addition, it also suggests that patients tend to endure symptoms and visit the hospital later in the pandemic era (25). This may be associated with the new policies formulated during the COVID-19 pandemic dealing with social distancing as well as patient fears about contracting the disease from the hospital. On the other hand, there is another opinion that the high rate of complicated appendicitis may also be attributed to the increased number of self-resolving simple or early appendicitis treated outside the hospital, rather than the actual increase in the number of severe cases due to delay in visiting a medical institution (26, 27). However, according to our results, the number of patients with acute appendicitis between the pre-pandemic and the pandemic period was not changed, and the proportion of complicated appendicitis increased. Furthermore, as TSH was significantly longer in the pandemic period, we suggested that the increased proportion of complicated appendicitis might have been attributed to prolonged TSH.

Other studies have also reported an increase in the number of complicated appendicitis cases during the pandemic compared to that during the pre-pandemic period (19, 23, 28, 29). These studies defined complicated appendicitis using various methods, such as pathological results, radiological findings, or surgical findings. A study by Orthopoulos et al. (19) defined complicated appendicitis based on pathological findings, while a study by Romero et al. (29) classified CT findings into five grades and defined a higher grade as higher severity. In another study, complicated appendicitis was defined according to intraoperative findings (23). However, discrepancies may exist between CT findings, surgical findings, and pathological results, and complicated appendicitis may not be included in each analysis. Therefore, complicated appendicitis should be classified by considering radiological, intraoperative, and pathological findings simultaneously, as done in the present study. Our criteria may reflect the complicated appendicitis criteria that surgeons use for classification in real-world practice.

Our results showed that the mean operation time has been shortened during the pandemic despite the increased rate of complicated appendicitis. These findings might have been attributed to the surgeons’ efforts to minimize the operation time to reduce possible contamination during the pandemic or because the characteristics of individual surgeons that performed operations during the study period were different.

Interestingly, as the COVID-19 pandemic situation worsened, that is, as the number of confirmed daily cases increases, the TSH was prolonged and the rate of complicated appendicitis increased. On the other hand, as the cumulative vaccination rate increased, the TSH and the proportion of complicated appendicitis decreased. The number of confirmed cases may negatively influence governmental policies for infection control and the social phobia of being infected with COVID-19. Therefore, hospital arrivals may be delayed, and the number of cases with complicated appendicitis may increase as the number of confirmed COVID-19 cases increases. The Korean government strongly recommended vaccination for individuals, and the vaccine was quickly distributed as a national infection control policy since the first vaccination was started on February 26, 2021. As the cumulative vaccination rate increased, the government’s social distancing policy gradually relaxed. In addition, as the proportion of those who were vaccinated increased, the fear of coronavirus infection in the hospital may have gradually decreased. Therefore, patients may feel comfortable visiting the ER without delay after symptom onset. Vaccination may be one of the factors contributing to a shortening of TSH.

While our study reported that the THO was not prolonged during the study period, other studies reported that the THO was prolonged due to the PCR test for COVID-19 screening (25, 30). In our institution, standard real-time reverse transcription-polymerase chain reaction (RT-PCR) was performed for patients who needed to be admitted to general wards or the intensive care unit (ICU) via the ER; however, Xpert Xpress SARS-Cov-2 (Cepheid, USA), which is an automated diagnostic test for the qualitative detection of nucleic acid from SARS-CoV-2, was performed for patients who needed emergency surgery. The standard RT-PCR takes about 6–8 h, while the Xpert test takes about 30 min to 1 h on average, with an excellent test performance (31). CT scans are used to diagnose acute appendicitis in Korea after checking creatinine levels. Because it took at least 30 to 60 min to report the serum creatinine level and the result of Xpert was normally reported within that time, the THO may not have increased in our study. The use of rapid PCR tests such as Xpert may help prevent time delay in the treatment of acute appendicitis.

In the present study, the proportion of patients who received antibiotic therapy with or without percutaneous drainage followed by interval appendectomy increased during the pandemic period, in particular, the 2^nd^ year of the pandemic (2021) compared with that during the pre-pandemic period (PP 2.9%, P-BV 1.6%, P-AV 10.7%, *P* = 0.06, data not shown). The incidence of interval appendectomy may be increased due to the increase in cases of complicated appendicitis resulting from delayed time from symptom onset to ER visit. According to previous studies, interval appendectomy was not inferior to the initial surgical approach in terms of reducing postoperative morbidity (16, 32). Although the initial surgical approach was still preferred in South Korea, interval appendectomy increased as the proportion of complicated appendicitis cases increased during the pandemic. However, conservative management as an initial treatment for acute appendicitis has been recommended in the UK during the COVID-19 pandemic era (17). The recommendation in the UK and the results of other studies showed that antibiotic application was also effective and non-inferior compared to the initial surgical approach (15, 33). A randomized controlled trial with a large number of patients suggested that initial antibiotic therapy was non-inferior to appendectomy based on symptom resolution and the results of a standard health status measure using the European Quality of Life-5 Dimensions (EQ-5D). However, care should be taken in interpretation because an appendectomy was performed in 29% of the antibiotic group within 90 days, and complications were more common in the antibiotic group in the study (15). In addition, Sceats et al. demonstrated that the non-operative management of acute appendicitis was associated with higher rates of abscess, readmission, and overall cost of care (14). Therefore, there is still a debate between the surgical approach and conservative management as initial treatment, and the change in the treatment algorithm for acute appendicitis in the UK could be a great challenge and an inevitable choice in the event of a medical resource shortage during the COVID-19 pandemic. Although it is not possible to determine which is correct because in- and out-of-hospital circumstances are different, some efforts to maintain the mainstream of previous practice, such as the use of a rapid PCR test and rapid distribution of vaccines, are necessary to maintain the quality of treatment for acute appendicitis.

This trend is not limited to appendicitis. A similar trend is observed in acute cholecystitis cases. The standard treatment for acute cholecystitis is laparoscopic cholecystectomy, and conservative treatment, such as percutaneous cholecystostomy, can be performed in patients in the high-risk group for surgery. However, during the pandemic period, percutaneous cholecystostomy was preferred, in order to avoid laparoscopic surgery, even if the patient was not in a poor general condition (34–36).

This study has several limitations. First, there was an inevitable selection bias due to its retrospective nature. Second, there may also be a bias due to the heterogeneous decision on treatment and surgical strategy for each surgeon. Third, because this study was conducted based on data from only single institution, it may not reflect the overall trend. Fourth, since the vaccination history of each patient could not be retrospectively confirmed with the medical record, a direct relationship between the actual vaccination history and disease characteristics could not be confirmed.

Despite these limitations, this study is meaningful because we confirmed the trend for 2 years during the pandemic. To our knowledge, this is the first study to include an analysis of the difference in outcomes before and after vaccination and to report the correlation of the number of daily confirmed cases and the cumulative vaccination rate with the outcomes. In addition, this study has an important strength because we classified complicated appendicitis by using radiological, intraoperative, and pathological findings simultaneously to adjust the discrepancies between the results.

In conclusion, patient hospital arrivals were delayed in the COVID-19 pandemic period compared to those in the pre-pandemic period. This result may be due to the fear of contracting COVID-19 and the government’s stringent social distancing policy. The proportion of complicated appendicitis during the pandemic was higher than that before the pandemic, which may be due to the delayed time to hospital arrival. In addition, with the increase in the number of daily confirmed cases, TSH has been prolonged, and the rate of complicated appendicitis has increased. On the other hand, with the increase in vaccination rate, the TSH decreased, and the rate of complicated appendicitis decreased, possibly due to relaxation of the social distancing policy and reduced fear of visiting the hospital. Following the ER visit, surgery for the patients was not delayed because we used a rapid PCR test for screening. Therefore, in-hospital efforts to avoid further time delay, such as applying rapid PCR tests, and nationwide efforts to shorten the TSH, such as vaccination and creating a social atmosphere in which people with symptoms do not hesitate to visit the hospital, are necessary to maintain the quality of treatment for acute appendicitis during an infectious disease pandemic.

## Data Availability

The raw data supporting the conclusions of this article will be made available by the authors, without undue reservation

## References

[1] Guan WJ Ni ZY Hu Y Liang WH Ou CQ He JX Clinical characteristics of coronavirus disease 2019 in China. N Engl J Med. (2020) 382:1708–20. 10.1056/NEJMoa2002032 32109013 PMC7092819

[2] Sohrabi C Alsafi Z O’Neill N Khan M Kerwan A Al-Jabir A World Health Organization declares global emergency: a review of the 2019 novel coronavirus (COVID-19). Int J Surg. (2020) 76:71–6. 10.1016/j.ijsu.2020.02.034 32112977 PMC7105032

[3] Gostin LO Wiley LF. Governmental public health powers during the COVID-19 pandemic: stay-at-home orders, business closures, and travel restrictions. *JAMA*. (2020) 323:2137–8. 10.1001/jama.2020.5460 32239184

[4] Cucinotta D Vanelli M. WHO declares COVID-19 a pandemic. Acta Biomed. (2020) 91:157–60. 10.23750/abm.v91i1.9397 32191675 PMC7569573

[5] Sung HK Paik JH Lee YJ Kang S. Impact of the COVID-19 outbreak on emergency care uilization in patients with acute myocardial infarction: a nationwide population-based study. J Korean Med Sci. (2021) 36:e111. 10.3346/jkms.2021.36.e111 33904263 PMC8076842

[6] Boserup B McKenney M Elkbuli A. The impact of the COVID-19 pandemic on emergency department visits and patient safety in the United States. Am J Emerg Med. (2020) 38:1732–6. 10.1016/j.ajem.2020.06.007 32738468 PMC7274994

[7] Englehardt RK Nowak BM Seger MV Duperier FD. Contamination resulting from aerosolized fluid during laparoscopic surgery. JSLS. (2014) 18. 10.4293/JSLS.2014.00361 25392644 PMC4154434

[8] Di Saverio S Khan M Pata F Ietto G De Simone B Zani E Laparoscopy at all costs? Not now during COVID-19 outbreak and not for acute care surgery and emergency colorectal surgery: a practical algorithm from a hub tertiary teaching hospital in Northern Lombardy, Italy. J Trauma Acute Care Surg. (2020) 88:715–8. 10.1097/TA.0000000000002727 32282750 PMC7473818

[9] Deng JZ Chan JS Potter AL Chen YW Sandhu HS Panda N The risk of postoperative complications after major elective surgery in active or resolved COVID-19 in the United States. Ann Surg. (2022) 275:242–6. 10.1097/SLA.0000000000005308 34793348 PMC8745943

[10] Nepogodiev D Bhangu A Glasbey JC Li E Omar OM Simoes JFF Mortality and pulmonary complications in patients undergoing surgery with perioperative SARS-CoV-2 infection: an international cohort study. Lancet. (2020) 396:27–38. 10.1016/s0140-6736(20)31182-x 32479829 PMC7259900

[11] Moletta L Pierobon ES Capovilla G Costantini M Salvador R Merigliano S International guidelines and recommendations for surgery during Covid-19 pandemic: a systematic review. Int J Surg. (2020) 79:180–8. 10.1016/j.ijsu.2020.05.061 32454253 PMC7245259

[12] Bhangu A Soreide K Di Saverio S Assarsson JH Drake FT. Acute appendicitis: modern understanding of pathogenesis, diagnosis, and management. Lancet. (2015) 386:1278–87. 10.1016/S0140-6736(15)00275-5 26460662

[13] Addiss DG Shaffer N Fowler BS Tauxe RV. The epidemiology of appendicitis and appendectomy in the United States. Am J Epidemiol. (1990) 132:910–25. 10.1093/oxfordjournals.aje.a115734 2239906

[14] Sceats LA Trickey AW Morris AM Kin C Staudenmayer KL. Nonoperative management of uncomplicated appendicitis among privately insured patients. JAMA Surg. (2019) 154:141–9. 10.1001/jamasurg.2018.4282 30427983 PMC6439669

[15] Collaborative C Flum DR Davidson GH Monsell SE Shapiro NI Odom SR A randomized trial comparing antibiotics with appendectomy for appendicitis. N Engl J Med. (2020) 383:1907–19. 10.1056/NEJMoa2014320 33017106

[16] Jo Y-S Yang S-S Im Y-C Park D-J Kim G-Y. Therapeutic consideration of periappendiceal abscess: an evaluation of non-surgical treatment followed by minimally invasive interval appendectomy. J Minim Invasive Surg. (2017) 20:129–36. 10.7602/jmis.2017.20.4.129

[17] Javanmard-Emamghissi H Boyd-Carson H Hollyman M Doleman B Adiamah A Lund JN The management of adult appendicitis during the COVID-19 pandemic: an interim analysis of a UK cohort study. Tech Coloproctol. (2021) 25:401–11. 10.1007/s10151-020-02297-4 32671661 PMC7362319

[18] Zheng Z Bi JT Liu YQ Cai X. The impact of COVID-19 pandemic on the treatment of acute appendicitis in China. Int J Colorectal Dis. (2022) 37:215–9. 10.1007/s00384-021-04031-4 34647160 PMC8514203

[19] Orthopoulos G Santone E Izzo F Tirabassi M Perez-Caraballo AM Corriveau N Increasing incidence of complicated appendicitis during COVID-19 pandemic. Am J Surg. (2021) 221:1056–60. 10.1016/j.amjsurg.2020.09.026 33012500 PMC7521886

[20] Kohler F Acar L van den Berg A Flemming S Kastner C Muller S Impact of the COVID-19 pandemic on appendicitis treatment in Germany-a population-based analysis. Langenbecks Arch Surg. (2021) 406:377–83. 10.1007/s00423-021-02081-4 33420517 PMC7794073

[21] Kearney D Cahill RA O’Brien E Kirwan WO Redmond HP. Influence of delays on perforation risk in adults with acute appendicitis. Dis Colon Rectum. (2008) 51:1823–7. 10.1007/s10350-008-9373-6 18584252

[22] COVID-19 Vaccine and Vaccination (2021). Available at: https://ncv.kdca.go.kr/ (Accessed December 1, 2021).

[23] Angeramo CA Dreifuss NH Schlottmann F Rotholtz NA. More severe presentations of acute appendicitis during COVID-19. J Gastrointest Surg. (2021) 25:1902–4. 10.1007/s11605-020-04892-0. 33469887 PMC7815199

[24] Zhang Y Chen YP Wang J Deng Y Peng D Zhao L. Anxiety status and influencing factors of rural residents in hunan during the coronavirus disease 2019 epidemic: a web-based cross-sectional survey. Front Psychiatry. (2020) 11:564745. 10.3389/fpsyt.2020.564745 33329105 PMC7732504

[25] Kim CW Lee SH. Impact of COVID-19 on the care of acute appendicitis: a single-center experience in Korea. Ann Surg Treat Res. (2021) 101:240–6. 10.4174/astr.2021.101.4.240 34692596 PMC8506019

[26] Chang YJ Chen LJ Chang YJ. Did the severity of appendicitis increase during the COVID-19 pandemic? PLoS One. (2022) 17:e0263814. 10.1371/journal.pone.0263814 35143582 PMC8830628

[27] Ielpo B Podda M Pellino G Pata F Caruso R Gravante G Global attitudes in the management of acute appendicitis during COVID-19 pandemic: ACIE Appy Study. Br J Surg. (2021) 108:717–26. 10.1002/bjs.11999 34157090 PMC7675377

[28] Antakia R Xanthis A Georgiades F Hudson V Ashcroft J Rooney S Acute appendicitis management during the COVID-19 pandemic: a prospective cohort study from a large UK centre. Int J Surg. (2021) 86:32–7. 10.1016/j.ijsu.2020.12.009 33465496 PMC7985094

[29] Romero J Valencia S Guerrero A. Acute appendicitis during coronavirus disease 2019 (COVID-19): changes in clinical presentation and CT findings. J Am Coll Radiol. (2020) 17:1011–3. 10.1016/j.jacr.2020.06.002 32610104 PMC7321660

[30] Lee KY Lee J Park YY Oh ST. Effect of the COVID-19 pandemic on surgical treatment of acute appendicitis: a single-center retrospective study. Asian J Surg. (2021) 44:800–1. 10.1016/j.asjsur.2021.03.012 33863628 PMC8081671

[31] Procop GW Brock JE Reineks EZ Shrestha NK Demkowicz R Cook E A comparison of five SARS-CoV-2 molecular assays with clinical correlations. Am J Clin Pathol. (2021) 155:69–78. 10.1093/ajcp/aqaa181 33015712 PMC7665304

[32] Mima K Miyanari N Itoyama R Nakao Y Kato R Shigaki H Interval laparoscopic appendectomy after antibiotic therapy for appendiceal abscess in elderly patients. Asian J Endosc Surg. (2020) 13:311–8. 10.1111/ases.12758 31621202

[33] Collard M Lakkis Z Loriau J Mege D Sabbagh C Lefevre JH Antibiotics alone as an alternative to appendectomy for uncomplicated acute appendicitis in adults: changes in treatment modalities related to the COVID-19 health crisis. J Visc Surg. (2020) 157:S33–S42. 10.1016/j.jviscsurg.2020.04.014 32362368 PMC7181971

[34] Somuncu E Kara Y Kizilkaya MC Bozdag E Yildiz ZB Ozkan C Percutaneous cholecystostomy instead of laparoscopy to treat acute cholecystitis during the COVID-19 pandemic period: single center experience. Ulus Travma Acil Cerrahi Derg. (2021) 27:89–94. 10.14744/tjtes.2020.69804 33394477

[35] Shakir T Matwala K Vasan A Karamanakos S. Percutaneous cholecystostomy for acute cholecystitis: a three-year single-centre experience including during COVID-19. Cureus. (2021) 13:e20385. 10.7759/cureus.20385 35036216 PMC8752374

[36] Martinez Caballero J Gonzalez Gonzalez L Rodriguez Cuellar E Ferrero Herrero E Perez Algar C Vaello Jodra V Multicentre cohort study of acute cholecystitis management during the COVID-19 pandemic. Eur J Trauma Emerg Surg. (2021) 47:683–92. 10.1007/s00068-021-01631-1 33742223 PMC7978438

